# Social Media and Black Maternal Health: The Role of Health Literacy and eHealth Literacy

**DOI:** 10.3928/24748307-20230614-01

**Published:** 2023-07

**Authors:** Nerissa George, Simone Reynolds, Rachel de Long, Marilyn Kacica, Rukhsana Ahmed, Jennifer Manganello

## Abstract

**Background:**

Black women experience greater maternal mortality and morbidity than White women. Although there are many causes of this disparity, providing more and better maternal health information to this population may be beneficial. Social media offers a way to easily and quickly disseminate information to empower and educate Black women about health during pregnancy.

**Objective:**

This study sought to identify social media use patterns to determine what sources Black women used to obtain information about pregnancy and to explore whether health literacy/eHealth literacy influence those patterns.

**Methods:**

This cross-sectional, nationally representative survey panel included 404 Black women. Health literacy was measured by the Single Item Literacy Screener, and eHEALS was used to measure eHealth literacy. We examined participants' social media activity, social media use, social media use for support, and sharing of pregnancy-related health information. Relationships between health literacy, eHealth literacy, and social media use were assessed.

**Key Results:**

Overall, 67.5% of participants had high health literacy, and the average eHealth literacy score was high (34.5). Most women (71.6%) reported using more than three social media accounts as a source for pregnancy information. Women with low health literacy searched social media for general and specific pregnancy health information, reported more social media use during pregnancy in general (*p* < .001), and more use of social media for giving and getting support (*p* = .003). Women with higher eHealth literacy were more likely to report more social media use (*r* = 0.107, *p* = .039) and often used social media to give and get support (*r* = 0.197, *p* = .0001). Women with high health literacy more often reported sharing the pregnancy information they found on social media with their nurse (χ^2^ = 7.068, *p* = .029), doula (χ^2^ = 6.878, *p* = .032), and childbirth educator (χ^2^ = 10.289, *p* = .006). Women who reported higher eHealth literacy also reported more often sharing the pregnancy information they found on social media with their doctor (*r* = 0.115, *p* = .030), nurse (*r* = 0.139, *p* = .001), coworkers (*r* = 0.160, *p* = .004), and family or friends (*r* = 0.201, *p* = .0001).

**Conclusion:**

Substantial numbers of Black women use social media to find pregnancy health information. Future studies should elicit more detailed information on why and how Black women use social media to obtain pregnancy information and support as well as what role health literacy and eHealth literacy may have on birth outcomes. [***HLRP: Health Literacy Research and Practice*. 2023;7(3):e119–e129.**]

## Plain Language Summary

This study aimed to identify Black women's sources for pregnancy information and to examine whether a relationship exists between health literacy/eHealth literacy and social media use. Findings support that Black women use social media for pregnancy information. Those with lower health literacy use social media more often and use social media more to share and to obtain pregnancy health information.

According to the Centers for Disease Control and Prevention (CDC), roughly 700 women in the United States die from pregnancy-related complications each year and for each woman who dies of pregnancy complications, at least 70 other women come close to death (CDC, 2019; CDC, 2020). Black women are 3 times more likely to die from pregnancy-related health issues than White women (CDC, 2019) and are more likely to suffer from maternal morbidity than their White counterparts (CDC, 2020; Howell et al., 2016). These disparities can be traced to numerous causes, including access to care, quality of care, implicit bias, and chronic disease prevalence (Howell, 2018). National birth delivery data have shown that Black women experience higher pregnancy-induced and chronic comorbidities such as chronic hypertension, asthma, placental disorders, gestational diabetes, pre-existing diabetes, and blood disorders than White women (Howell et al., 2016). Chronic diseases associated with an increased risk for pregnancy-related mortality are more prevalent and less well-controlled among Black women, putting them at a higher risk for complications (Fryar et al., 2017).

Deficiencies in access to information and support have influenced health disparities. Significant gaps in the access and use of health information online are linked to income, education, and race and ethnicity (Gilmour, 2007). Social media information can be presented in many formats, including text, videos, and images, which can deliver health information to audiences with a range of needs, including those with lower health literacy. Social media widens access for everyone, but especially individuals who may not easily access health information via traditional methods, such as younger people, racial and ethnic populations, and lower socioeconomic groups (Moorhead et al., 2013); it has also demonstrated that it aids in changing health behavior (Chen & Wang, 2021). Individuals have used social networking sites to discuss health-related issues and complex information with health professionals (Colineau & Paris, 2010). Baker and Yang (2018) found that 43% of mothers within their sample used blogs to communicate with other mothers, 89% used social media sites for questions and advice related to pregnancy and their role as a parent, and 84% deemed social media friends a form of social support. Also, a meta-analysis found social media to effectively promote pregnancy weight control, gestational diabetes control, maternal well-being, and increasing pregnancy knowledge (Chan & Chen, 2019). However, very little information is available on how Black women use social media during pregnancy.

Although social media can be an effective channel to disseminate health information, it is also important to note that due to the lack of formal oversight on social media, misinformation can easily spread among users. A systematic review of misinformation on social media showed that misinformation was most prevalent on Twitter and most often occurred with health topics related to smoking products, drugs, vaccines, and diseases (Suarez-Lledo & Alvarez-Galvez, 2021). It is important to prevent or reduce the dissemination of misinformation whenever possible due to the ability of misinformation to influence. Not all social media users possess the needed skills to evaluate whether a post is of high quality or contains inaccurate information. A systematic review of the uses, benefits, and limitations of social media for health communication found that more social media users were Black compared to their White counterparts, belonged to lower-income households, and were women (Moorhead et al., 2013). In 2012, approximately 72% of U.S. adult internet users searched online for health information (Fox & Duggan, 2013). As of April 2021, 91% of Black adults reported using the internet, with 77% of Black adults using social media (Pew Research Center, 2021). Black women have high ownership of mobile devices (80% own smartphones and 57% own a tablet), providing access to the internet, social media, and phone apps (Nielsen Company, 2017). Black women spend more time weekly using apps and browsing the web on smartphones (19 hours and 27 minutes on average) than other women (17 hours and 8 minutes on average) (Nielsen Company, 2017). However, whether and how Black women use social media for maternal health information and support remain unclear. Despite the critical role social media can play in maternal health (Chan & Chen, 2019), only one study has examined social media use for health information and social support by Black women who are pregnant/postpartum (Asiodu et al., 2015).

Health literacy measures the ability to “obtain, process, understand and communicate about health-related information needed to make informed decisions” (Morris et al., 2006) and can affect a pregnant person's ability to find, comprehend, and use health information and make informed health decisions for themselves and the baby (Olander et al., 2018; Renkert & Nutbeam, 2001). eHealth literacy is “the ability to seek, find, understand, and appraise health information from electronic sources and apply the knowledge gained to address or solve a health problem”: it empowers and enables individuals to participate in health decisions informed by eHealth resources fully (Norman & Skinner, 2006). According to the National Assessment of Adult Literacy, only 12% of U.S. adults are proficiently health literate (Kutner, 2006). Higher health literacy plays a role in yielding positive pregnancy health outcomes (Kohan et al., 2007; Ohnishi et al., 2005). Currently, no prior work has examined the role of eHealth literacy in pregnancy health outcomes.

Both health literacy and eHealth literacy skills can also impact online health information seeking (Paige et al., 2017; Manganello et al., 2016), yet this relationship has not been widely studied for Black pregnant women. Limited health literacy has been associated with unhealthy behaviors during pregnancy among all women (Nawabi et al., 2021). Chen et al. (2018) found that lower health literacy is associated with lower chances of using medical websites for health information and higher odds of using television, social media, blogs, or celebrity webpages (Paige et al., 2017). They also found that individuals with lower health literacy used and relied on social media and blogs containing lower quality health information than health-care professionals' information (Chen et al., 2018). Currently, there are no existing studies on the association of eHealth literacy with maternal health outcomes or whether eHealth literacy is associated with the use of online health information among Black women, specifically for maternal health. The current high usage of social media for health information among pregnant people combined with Black women's vast access to social media displays that assessing eHealth literacy can enlighten researchers on a community's needs to find high-quality information on social media confidently.

Given the potential role of social media in informing Black women about maternal health and the lack of research on this topic, this project aimed to investigate the potential relationship between health literacy, eHealth literacy, and social media use for pregnancy-related topics among Black women. Because the intersection of social media, health literacy, and eHealth literacy may be an important component of reducing maternal health disparities, we investigated the following research questions:

1. What sources of information are most often used by Black women for pregnancy-related information?
2. What pregnancy-related topics do Black women most frequently seek on social media sites?
3. What is the association between health literacy and social media use during pregnancy?
4. What is the association between eHealth literacy and social media use during pregnancy?

## Methods

This study used a cross-sectional survey design. Although health disparities affect women of different races and ethnicities, given the continuously growing Black maternal health disparities within the U.S., combined with the known negative effect that low literacy has on health outcomes (Berkman et al., 2011), this study's target population was Black women. The University at Albany's Institutional Review Board approved this study (Protocol Number: 20X178).

### Recruitment

Survey data were collected from participants in a panel maintained by CloudResearch, a third-party online participant recruitment company. CloudResearch oversaw participant incentives. The survey was programmed into Qualtrics and pilot-tested for errors by the University at Albany research team and our CloudResearch assigned contact. Once both parties agreed to launch the survey, CloudResearch distributed the live survey link to eligible participants based on the information available on their database. When eligible participants clicked on the Qualtrics survey link, they were immediately directed to an eligibility screener. If participants met the inclusion criteria, they were taken to the informed consent page and asked to consent to the study.

### Survey

Data collection occurred from November 27, 2020 to December 4, 2020. Eligible participants self-identified as Black women, were at least age 18 years, spoke fluent English, resided in the United States, and were currently pregnant or had given birth within 12 months. The survey consisted of 41 questions and took about 15 minutes to complete. It was also piloted with 20 participants prior to launching data collection. The survey sought to detect social media use patterns of Black women during their most recent pregnancy. Questions also assessed the communication of social media information with each participant's networks. The appendix includes a copy of the survey questions used. Participants' demographics and clinical characteristics were collected as well. Key variables and measures are described below.

***Demographics.*** We collected each participant's age, education level, income, employment status, ethnicity, marital status, and the number of parents in the household. We also collected self-reported clinical characteristics such as a history of pregnancy complications, pregnancy status, number of live births, and type of pregnancy provider.

***Health literacy.*** The Single Item Literacy Screener (SILS) was used to measure health literacy. SILS is a simple validated instrument designed to identify patients with limited reading ability who need help reading health-related materials (Morris et al., 2006). The screener inquires, “How often do you need to have someone help you when you read instructions, pamphlets, or other written materials from your doctor or pharmacy?” Possible responses were 1 = *never*, 2 = *rarely*, 3 = *sometimes*, 4 = *often*, and 5 = *always*. Scores greater than 2 were considered lower health literacy, indicating difficulty reading printed health-related material.

***eHealth Literacy.*** The eHEALS scale was used to measure eHealth literacy. eHEALS is an 8-item validated scale intended to measure consumers' combined knowledge, comfort, and perceived skills to find, evaluate, and apply electronic health information to health problems (Norman & Skinner, 2006). Each item in the eHEALS uses a 5-point Likert scale to answer each question with response options ranging from 1 = *strongly disagree* to 5 = *strongly agree*. We modified the scale's wording from “health information” to “pregnancy information” to alleviate confusion with the questions. A total score was calculated based on respondents' answers; higher scores indicate higher eHealth literacy.

### Social Media

***Social media activity.*** Social media activity was measured by what social media platform women had a profile on and the frequency of use for each an (i.e., Facebook, Twitter, Instagram) on a 6-point Likert scale of 1 = *never* to 6 = *several times a day*. Scores less than 5 indicated no frequent use of respective social media platforms. Individuals were also asked to provide their best estimates for how often they used social media during their most recent pregnancy to obtain information on certain general and specific pregnancy-related topics selected by the research team. Responses were collected on a 5-point Likert scale from 1 = *never* to 5 = *always*.

We also assessed the usefulness of the pregnancy information participants found on social media by asking them, “how useful has the pregnancy information found on social media sites been to help you make decisions about your pregnancy?” Responses were collected on a 4-point Likert scale from 1 = *not useful at all* to 4 = *very useful*.

**Figure 1.**
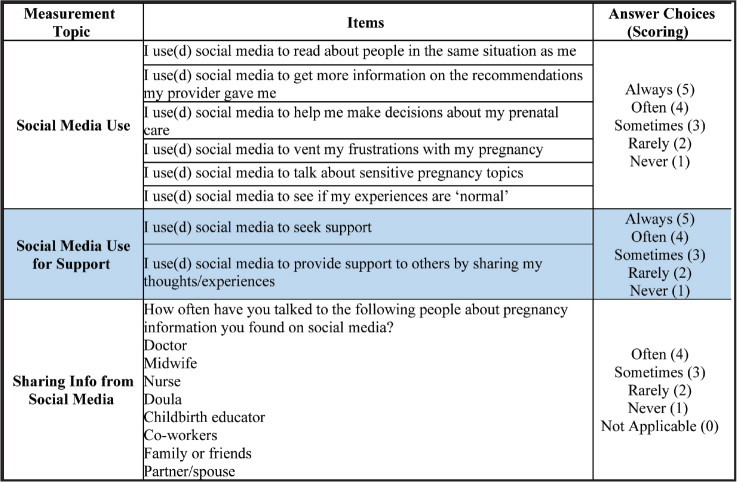
Social media use measurement instrument.

***Social media use.*** The frequency of the nature of social media use during pregnancy was measured by six adapted items **(Figure 1**). Questions were used from prior surveys in the existing pregnancy information-seeking literature (Bjelke et al., 2016; Hether et al., 2016). Some questions were modified as needed to improve readability for participants. Response options were collected using a 5-point Likert scale ranging from 1 = *never* to 5 = *always*. A total score was calculated by combining the responses from all six items; higher scores indicated more social media use.

***Social media use for support.*** Participants were asked to indicate how often they used social media for getting and giving support. Support was measured using two prior items. Questions **(Figure 1)** were utilized from prior surveys in the existing use of support during pregnancy literature (Bjelke et al., 2016; Hether et al., 2016). Response options were collected using a 5-point Likert scale ranging from 1 = *never* to 5 = *always*. A total score was calculated by combining the responses for both items; higher scores indicated more social media use for giving and getting support.

***Sharing pregnancy information found on social media.*** Participants were asked to indicate how often they shared the pregnancy information they found on social media with eight different individuals in their lives. Questions **(Figure 1)** were used from a prior study (Dekker et al., 2016). Responses were collected using a 5-point Likert scale where 0 = *not applicable*, 1 = *never*, 2 = *rarely*, 3 = *sometimes*, and 4 = o*ften*. *Not applicable* responses were excluded from the analysis. Separate analyses were conducted for each channel/individual participants reported sharing the information they found on social media.

### Statistical Analysis

Descriptive statistics were calculated for continuous (means and standard deviations) and categorical variables (frequencies and %). Independent sample *t*-tests, Pearson correlation, and chi-squares were performed to assess bivariate relationships between dependent (demographics and social media use) and independent variables (eHealth literacy and health literacy) when appropriate. Significance of alpha = 0.05 was used, and all tests were two-sided. Regression models were used to explore further whether observed bivariate interactions remained significant when controlling for up to four demographic characteristics: education level, age, the occurrence of pregnancy complications, and income. All demographic characteristics except age and pregnancy complications (due to its binary response option) were coded as dummy variables. All analyses were completed using SAS version 9.2.

## Results

**Figure 2.**
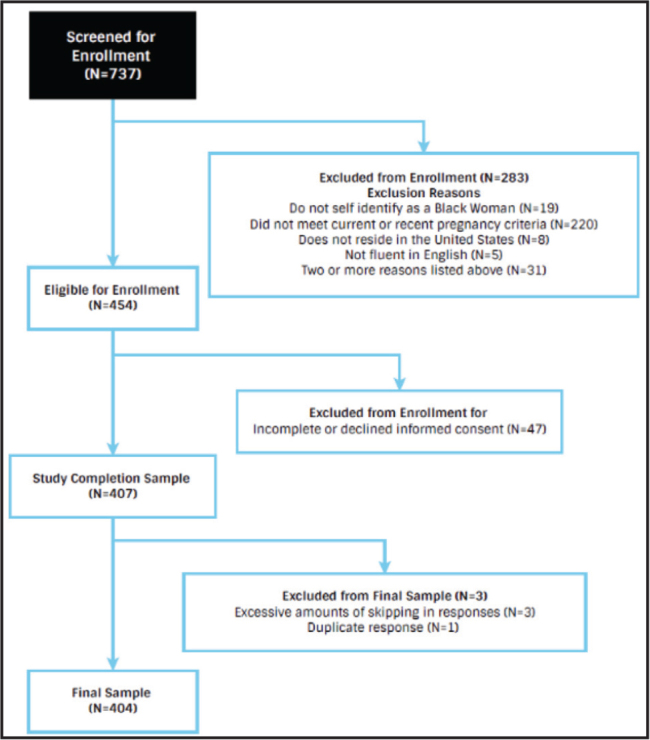
Study eligibility flow chart.

The final sample consisted of 404 Black women **(Figure 2)**. Within the sample, 52.7% were pregnant at study completion **(Table 1)**. Most (78.7%) women within the sample had at least one pregnancy before joining the study. The average age was 26.4 years, ranging from 18 to 40 years. Approximately 49.1% of the sample reported household income was less than $40,000. More than one-half (66.8%) of this sample indicated they were employed (full-time or part-time). This sample's education levels were diverse; **Table 1** displays the remaining demographics. This sample also had adequate health literacy (67.5%; SILS scores *of* < 2) and high eHealth literacy (mean eHEALS score = 34.5, with a range from 19 to 40). Women with adequate health literacy had higher eHealth literacy scores than women with low health literacy (34.9 vs. 33.7, *p* = .033, respectively). On average social media use scores in this study indicated that about 67.6% used social media for information seeking (mean scores were 20.27 out of 30), and roughly 65.9% used social media for giving and getting support (mean scores were 6.59 out of 10). Results also indicate that, on average, women mostly shared the pregnancy information they found on social media with their partner/spouse (mean score of 3.28 out of 4), family or friends (mean score of 3.14 out of 4), and their doctor (mean score of 3.04 out of 4).

**Table 1:**
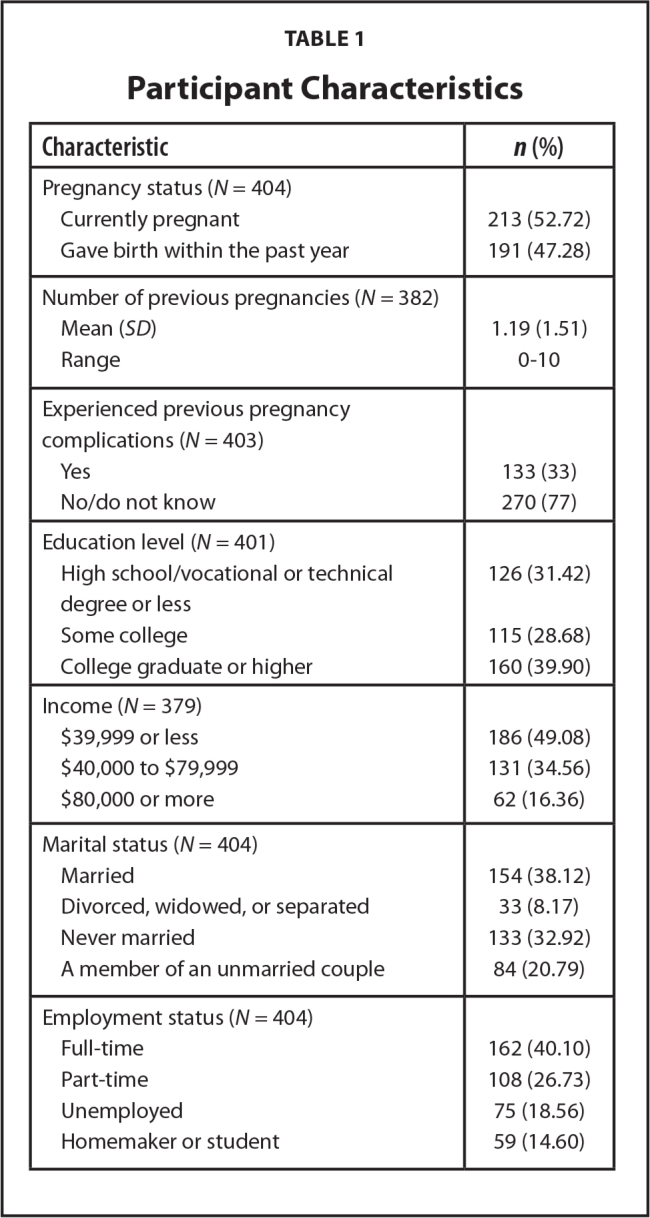
Participant Characteristics

**Characteristic**	***n* (%)**

Pregnancy status (*N* = 404)	
Currently pregnant	213 (52.72)
Gave birth within the past year	191 (47.28)

Number of previous pregnancies (*N* = 382)	
Mean (*SD*)	1.19 (1.51)
Range	0–10

Experienced previous pregnancy complications (*N* = 403)	
Yes	133 (33)
No/do not know	270 (77)

Education level (*N* = 401)	
High school/vocational or technical degree or less	126 (31.42)
Some college	115 (28.68)
College graduate or higher	160 (39.90)

Income (*N* = 379)	
$39,999 or less	186 (49.08)
$40,000 to $79,999	131 (34.56)
$80,000 or more	62 (16.36)

Marital status (*N* = 404)	
Married	154 (38.12)
Divorced, widowed, or separated	33 (8.17)
Never married	133 (32.92)
A member of an unmarried couple	84 (20.79)

Employment status (*N* = 404)	
Full-time	162 (40.10)
Part-time	108 (26.73)
Unemployed	75 (18.56)
Homemaker or student	59 (14.60)

### Sources of Information Use

Results suggested that nearly everyone in the sample owned or had access to a smartphone (99.5%). Approximately 85.4% of the women in this sample indicated that they had a Facebook account, 60.9% a Twitter account, and 89.8% an Instagram account. During their most recent pregnancy, 71.6% indicated they used at least three social media sites to find pregnancy information. Besides using social media, the women within this sample indicated they *always* or *often used* their family (77.1%), friends (57.6%), and pregnancy-related courses (47.4%) as a source for pregnancy information.

### Pregnancy-Related Information Sought

More than 50% of all Black women in this sample always or often searched social media for general pregnancy information regarding their bodies during pregnancy, sleep-related issues, and personal care products safe for use during pregnancy. Roughly 60% of all the women within this sample always or often searched social media for pregnancy information related to prenatal care. More than 60% of the entire sample searched social media for pregnancy information related to fetal growth, development, and delivery. See **Table 2** for the remaining results of topics Black women searched on social media. About 81.9% of the entire sample found pregnancy information on social media sites *useful* or *very useful* to help them make pregnancy decisions. Those who indicated that the pregnancy information they found on social media was *useful* to help them make pregnancy decisions were more likely to have higher eHealth literacy (mean difference = 1.302, *p* = .050) and health literacy than those who mentioned the information was not useful (χ^2^ = 4.556, *p* = .033).

**Table 2:**
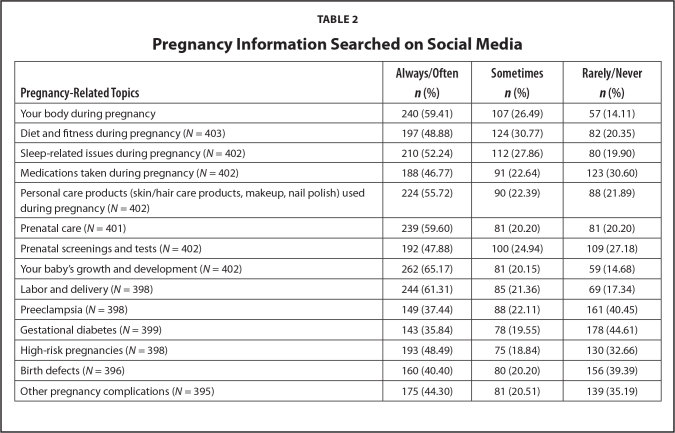
Pregnancy Information Searched on Social Media

**Pregnancy-Related Topics**	**Always/Often *n* (%)**	**Sometimes *n* (%)**	**Rarely/Never *n* (%)**
Your body during pregnancy	240 (59.41)	107 (26.49)	57 (14.11)
Diet and fitness during pregnancy (*N* = 403)	197 (48.88)	124 (30.77)	82 (20.35)
Sleep-related issues during pregnancy (*N* = 402)	210 (52.24)	112 (27.86)	80 (19.90)
Medications taken during pregnancy (*N* = 402)	188 (46.77)	91 (22.64)	123 (30.60)
Personal care products (skin/hair care products, makeup, nail polish) used during pregnancy (*N* = 402)	224 (55.72)	90 (22.39)	88 (21.89)
Prenatal care (*N* = 401)	239 (59.60)	81 (20.20)	81 (20.20)
Prenatal screenings and tests (*N* = 402)	192 (47.88)	100 (24.94)	109 (27.18)
Your baby's growth and development (*N* = 402)	262 (65.17)	81 (20.15)	59 (14.68)
Labor and delivery (*N* = 398)	244 (61.31)	85 (21.36)	69 (17.34)
Preeclampsia (*N* = 398)	149 (37.44)	88 (22.11)	161 (40.45)
Gestational diabetes (*N* = 399)	143 (35.84)	78 (19.55)	178 (44.61)
High-risk pregnancies (*N* = 398)	193 (48.49)	75 (18.84)	130 (32.66)
Birth defects (*N* = 396)	160 (40.40)	80 (20.20)	156 (39.39)
Other pregnancy complications (*N* = 395)	175 (44.30)	81 (20.51)	139 (35.19)

### Health Literacy

Women who had low health literacy were more likely to use social media to obtain information on their body during pregnancy (mean difference = 0.343, *p* = .004), diet and fitness (mean difference = 0.297, *p* = .020), medications taken during pregnancy (mean difference = 0.306, *p* = .027), personal care products safe to use during pregnancy (mean difference = 0.319, *p* = .024), prenatal screenings and testing (mean difference = 0.338, *p* = .013), delivery (mean difference = 0.331, *p* = .010), pre-eclampsia (mean difference = 0.491, *p* = .002), and gestational diabetes (mean difference = 0.485, *p* = .002) compared to women with higher health literacy. Women with low health literacy reported more social media use (mean difference = 2.028, *p* < .001) and more social media use for giving and getting support (mean difference = 0.726, *p* = .003; see **Table 3**) than women with high health literacy. Women with higher health literacy more often reported sharing the pregnancy information they found on social media with their nurse (χ^2^ = 7.068, *p* = .029), doula (χ^2^ = 6.878, *p* = .032), and childbirth educator (χ^2^ = 10.289, p =0.006) than women with lower health literacy. Regardless of health literacy, the social media activity was the same across platforms such as Facebook (χ^2^ = 1.052, *p* = .305), Twitter (χ^2^ = 0.838, *p* = .360), and Instagram (χ^2^ = 0.0571, *p* = .811). Regression analyses showed the relationships between lower health literacy and social media use (F(4,387) = 4.75, *p* = .001) and social media use for getting and giving support (F(4,386) = 4.45, *p* = .002) during pregnancy remained significant when controlling for age and education. See **Table 4** for a visual representation of the models. Therefore, lower health literacy increased these Black women's likelihood of using social media and made them more inclined to use social media to give and get pregnancy support.

**Table 3:**
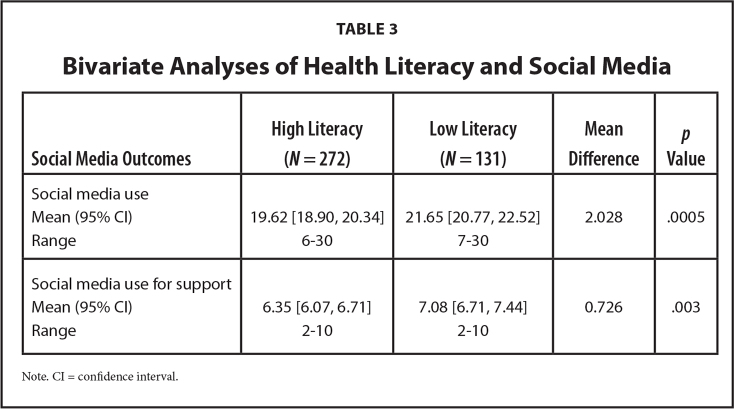
Bivariate Analyses of Health Literacy and Social Media

**Social Media Outcomes**	**High Literacy (*N* = 272)**	**Low Literacy (*N* = 131)**	**Mean Difference**	***p* Value**

Social media use				
Mean (95% CI)	19.62 [18.90, 20.34]	21.65 [20.77, 22.52]	2.028	.0005
Range	6–30	7–30		

Social media use for support				
Mean (95% CI)	6.35 [6.07, 6.71]	7.08 [6.71, 7.44]	0.726	.003
Range	2–10	2–10		

Note. CI = confidence interval.

**Table 4:**
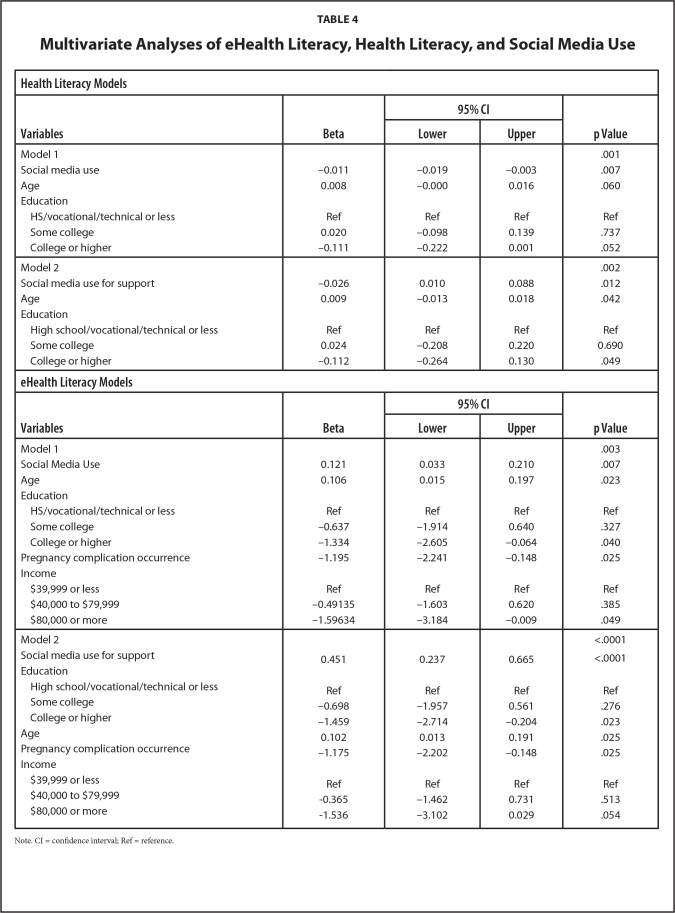
Multivariate Analyses of eHealth Literacy, Health Literacy, and Social Media Use

**Health Literacy Models**				

**Variables**	**Beta**	**95% CI**		**p Value**

		**Lower**	**Upper**	

Model 1				.001
Social media use	−0.011	−0.019	−0.003	.007
Age	0.008	−0.000	0.016	.060
Education				
HS/vocational/technical or less	Ref	Ref	Ref	Ref
Some college	0.020	−0.098	0.139	.737
College or higher	−0.111	−0.222	0.001	.052

Model 2				.002
Social media use for support	−0.026	0.010	0.088	.012
Age	0.009	−0.013	0.018	.042
Education				
High school/vocational/technical or less	Ref	Ref	Ref	Ref
Some college	0.024	−0.208	0.220	0.690
College or higher	−0.112	−0.264	0.130	.049

**eHealth Literacy Models**				

**Variables**	**Beta**	**95% CI**		**p Value**

		**Lower**	**Upper**	

Model 1				.003
Social Media Use	0.121	0.033	0.210	.007
Age	0.106	0.015	0.197	.023
Education				
HS/vocational/technical or less	Ref	Ref	Ref	Ref
Some college	−0.637	−1.914	0.640	.327
College or higher	−1.334	−2.605	−0.064	.040
Pregnancy complication occurrence	−1.195	−2.241	−0.148	.025
Income				
$39,999 or less	Ref	Ref	Ref	Ref
$40,000 to $79,999	−0.49135	−1.603	0.620	.385
$80,000 or more	−1.59634	−3.184	−0.009	.049

Model 2				<.0001
Social media use for support	0.451	0.237	0.665	<.0001
Education				
High school/vocational/technical or less	Ref	Ref	Ref	Ref
Some college	−0.698	−1.957	0.561	.276
College or higher	−1.459	−2.714	−0.204	.023
Age	0.102	0.013	0.191	.025
Pregnancy complication occurrence	−1.175	−2.202	−0.148	.025
Income				
$39,999 or less	Ref	Ref	Ref	Ref
$40,000 to $79,999	−0.365	−1.462	0.731	.513
$80,000 or more	−1.536	−3.102	0.029	.054

Note. CI = confidence interval; Ref = reference.

### eHealth Literacy

Our results suggest that higher eHealth literacy increases the likelihood of women using social media to obtain general pregnancy information on their body during pregnancy (*r* = 0.118, *p* = .024), diet and fitness (*r* = 0.188, *p* < .001), sleep-related issues (*r* = 0.182, *p* < .001), medications taken during pregnancy (*r* = 0.138,*p* = .008), personal care products to use during pregnancy (*r* = 0.110, *p* = .035), and specific pregnancy topics such as prenatal screenings and testing (*r* = 0.128, *p* = .014), delivery (*r* = 0.155, *p* = .003), and their baby development/growth (*r* = 0.222, *p* < .0001). We found that women with higher eHealth literacy were more likely to report more social media use (*r* = 0.107, *p* = .039) and give and get support more from social media (*r* = 0.197, *p* = .0001) during pregnancy (see **Table 5**). Women who reported higher eHealth literacy also reported more often sharing the pregnancy information they found on social media with their doctor (*r* = 0.115, *p* = .030), nurse (*r* = 0.139, *p* = .001), coworkers (*r* = 0.160, *p* = .004), and family or friends (*r* = 0.201, *p* = .0001). Women who indicated frequent Facebook use often had significantly higher eHealth literacy than those who did not use Facebook often (mean eHEALS score 35.07 vs. 33.46, *p* = .006, respectively). However, there was no notable difference in eHealth literacy scores amongst those who reported frequent use of Instagram or Twitter. Separate regression models revealed that after controlling for age, education, the experience of pregnancy complications, and income, women with higher eHealth literacy were still more likely to report using social media (F(7,353) = 3.14, *p* = .003) and using social media for getting and giving support (F(7,352) = 4.61, *p* = .00268, *p* < .0001) during pregnancy. Therefore, higher eHealth literacy increased these Black women's likelihood of using social media and made them more inclined to use social media to give and get pregnancy support. See **Table 4** for a visual representation of the models.

**Table 5:**
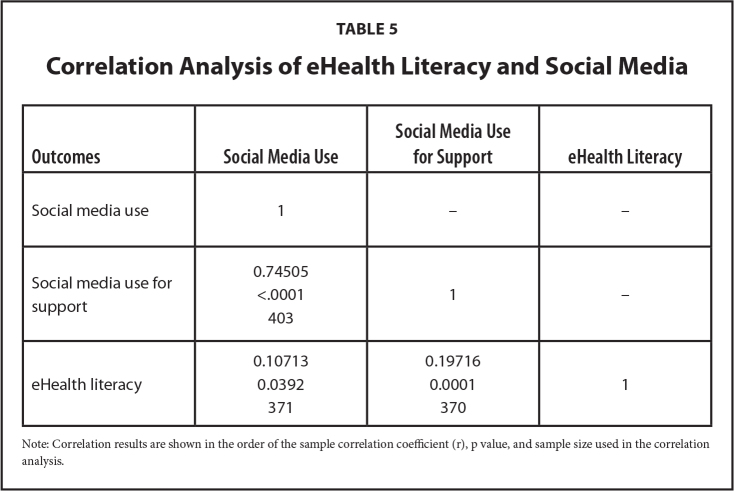
Correlation Analysis of eHealth Literacy and Social Media

**Outcomes**	**Social Media Use**	**Social Media Use for Support**	**eHealth Literacy**

Social media use	1	–	–

Social media use for support	0.74505	1	–
	<.0001		
	403		

eHealth literacy	0.10713	0.19716	1
	0.0392	0.0001	
	371	370	

Note: Correlation results are shown in the order of the sample correlation coefficient (r), p value, and sample size used in the correlation analysis.

## Discussion

This study focused on Black women in the U.S. because existing literature lack key details regarding their social media use during pregnancy to inform social media maternal health interventions. Study results confirm that Black women within this sample use social media as a source for pregnancy-related health information, to seek pregnancy information, give and get support, and share pregnancy-related information. Also, most women used informal sources such as social media, their family, and friends to obtain pregnancy information. Black pregnant women with low health literacy used social media more overall and were more inclined to use social media to give and get support during pregnancy. Black women with higher health literacy more often reported sharing information online with others during their pregnancy than those with low health literacy. It was also observed that Black birthing women with higher eHealth literacy were more likely to report using social media, giving and getting support from social media, and often shared pregnancy information they found on social media with others.

Several prior studies have examined health literacy in pregnant women and found that the prevalence of low health literacy ranged between 15% to 30% (Ghanbari et al., 2012; Shieh et al., 2009; Yee et al., 2021). Our sample's low health literacy was close to their range (32.5%). This study found that Black women with low health literacy were more likely to use social media and used social media more often for getting and giving support during pregnancy. This might be due to these women's need for on-demand accessible supplemental aid to understand their physical and mental health needs during pregnancy. Therefore, researchers and program developers need to create information and interventions that improve the quality of maternal health education and communication that reaches this population. Individuals exposed to false information who possess lower health literacy and poor analytical skills may be unable to evaluate the accuracy of online information, especially found on social media, effectively. Health literacy interventions that enable an individual to further enhance their skills in accessing, understanding, analyzing, and applying health information are needed. Pregnancy is a period when women benefit significantly from engaging in health services and are considered open to learning health-related information and behaviors, leading to opportunities to increase their health literacy (Crozier et al., 2009; Ghanbari et al., 2012; Olander et al., 2018). It is also important to note that health literacy encompasses more than the readability of health information, which was the measure used in this study. Future research should examine other components of health literacy such as the ability to find health information, comprehend health information, and use health information and health services. Other measures of health literacy can be utilized to get a more complete picture.

To our knowledge, this is also one of the first studies to measure eHealth literacy in a sample of pregnant Black women. Brewer et al. (2018) used the eHEALS scale on Black women, and our findings of eHealth literacy among this population were consistent. Our sample had high eHealth literacy, and our findings suggest that women with high eHealth literacy are more likely to report more social media use and give and get support from social media often during pregnancy than women with lower eHealth literacy. This observed relationship might be due to this sample's often usage of social media and age range, which may influence their comfortability with social media.

Very limited studies have examined knowledge levels regarding pregnancy complications among pregnant women in the U.S., especially among pregnant Black women. Studies have found that women's perceived knowledge about pregnancy complications tends to be higher than their actual knowledge of pregnancy complications (Sheinis et al., 2018), and significant knowledge deficiencies regarding common and severe health hazards associated with pregnancy persist despite education (Mellon et al., 2020). Education and support can play significant roles in reducing disparities, increasing awareness of maternal health along with their ability to identify warning signs of pregnancy complications (Neighmond, 2019). Using the findings from this study to guide the creation of high-quality evidence-based maternal health content that educate and support pregnant women, especially Black women, can help reduce negative pregnancy outcomes. Social media can provide an opportunity to disseminate this information at an affordable price (Huesch et al., 2016). Recent federal legislation intends to increase low-income households' access to the internet, providing more opportunities for all to use social media to provide health-related information. Combining this attempt to address the digital divide with properly equipping individuals with access and the needed skills to assess the quality and credibility (Taheri et al., 2021) would contribute to efforts to reduce maternal health disparities.

## Study Limitations

This cross-sectional design does not allow for any causal inferences. This project was subjected to the possible occurrence of recall bias; since we allowed Black women who recently gave birth within the past year to join the study, it might have been difficult for them to precisely remember the sources they used during their pregnancy. Future studies can limit their study criteria to people who are currently pregnant to reduce this bias. Although SILS is a validated measure, it can be considered a limited measure of health literacy because it only asks about one aspect of health literacy through one question. Future studies should examine additional parameters related to social media serving as a source of information such as trustworthiness and source credibility. Finally, our sample was recruited from an online third-party survey company; therefore, participants' willingness to engage in research and savviness to use technology may have resulted in a biased sample. This issue may influence our external validity because our observed associations might not accurately represent the general Black pregnant woman population. Future studies can recruit non-survey panel participants.

## Conclusion

A substantial number of Black women in our sample had lower health literacy. With our new knowledge regarding the link between low health literacy and higher social media use during pregnancy, developing tools or interventions that improve health literacy skills among this population is needed. Although health literacy is important, eHealth literacy skills development would be useful for enhancing and increasing social media use. Taking Black women's social media use patterns into account could be important for tailoring pregnancy health education and promoting health literacy, developing organizational health policy, and allocating resources for this population. Medical providers can make recommendations through social media accounts that use plain language and share high-quality, evidence-based pregnancy information. Future studies should consider how to elicit more detailed information on why Black women use social media often for information seeking and support during pregnancy. Also, future work can assess how Black women assess the quality and credibility of the information they encounter on social media and if they possess the skills needed to identify misinformation.

## References

[x24748307-20230614-01-bibr1] Asiodu , I. V. , Waters , C. M. , Dailey , D. E. , Lee , K. A. , & Lyndon , A. ( 2015 ). Breastfeeding and use of social media among first-time African American mothers . *Journal of Obstetric, Gynecologic, and Neonatal Nursing* , *44* ( 2 ), 268 – 278 10.1111/1552-6909.12552 PMID: 25712127 PMC4359664

[x24748307-20230614-01-bibr2] Baker , B. , & Yang , I. ( 2018 ). Social media as social support in pregnancy and the postpartum . *Sexual & Reproductive Healthcare : Official Journal of the Swedish Association of Midwives* , *17* , 31 – 34 10.1016/j.srhc.2018.05.003 PMID: 30193717

[x24748307-20230614-01-bibr3] Berkman , N. D. , Sheridan , S. L. , Donahue , K. E. , Halpern , D. J. , Viera , A. , Crotty , K. , Holland , A. , Brasure , M. , Lohr , K. N. , Harden , E. , Tant , E. , Wallace , I. , & Viswanathan , M . ( 2011 ). Health literacy interventions and outcomes: An updated systematic review . *Evidence Report/technology Assessment* , ( 199 ), 1 – 941 . PMID: 23126607 PMC4781058

[x24748307-20230614-01-bibr4] Bjelke , M. , Martinsson , A. K. , Lendahls , L. , & Oscarsson , M. ( 2016 ). Using the Internet as a source of information during pregnancy - A descriptive cross-sectional study in Sweden . *Midwifery* , *40* , 187 – 191 10.1016/j.midw.2016.06.020 PMID: 27450590

[x24748307-20230614-01-bibr5] Brewer , L. C. , Jenkins , S. , Lackore , K. , Johnson , J. , Jones , C. , Cooper , L. A. , Radecki Breitkopf , C. , Hayes , S. N. , & Patten , C. ( 2018 ). mHealth intervention promoting cardiovascular health among African Americans: Recruitment and baseline characteristics of a pilot study . *JMIR Research Protocols* , *7* ( 1 ), e31 10.2196/resprot.8842 PMID: 29386174 PMC5812978

[x24748307-20230614-01-bibr6] Centers for Disease Control and Prevention . ( 2019 ). *Pregnancy-related deaths* . https://www.cdc.gov/vitalsigns/maternal-deaths/index.html

[x24748307-20230614-01-bibr7] Centers for Disease Control and Prevention . ( 2020 ). *Severe maternal morbidity in the United States* . https://www.cdc.gov/reproductive-health/maternalinfanthealth/severematernalmorbidity.html

[x24748307-20230614-01-bibr8] Chan , K. L. , & Chen , M. ( 2019 ). Effects of social media and mobile health apps on pregnancy care: meta-analysis . *JMIR mHealth and uHealth* , *7* ( 1 ), e11836 10.2196/11836 PMID: 30698533 PMC6372934

[x24748307-20230614-01-bibr9] Chen , X. , Hay , J. L. , Waters , E. A. , Kiviniemi , M. T. , Biddle , C. , Schofield , E. , Li , Y. , Kaphingst , K. , & Orom , H. ( 2018 ). Health literacy and use and trust in health information . *Journal of Health Communication* , *23* ( 8 ), 724 – 734 10.1080/10810730.2018.1511658 PMID: 30160641 PMC6295319

[x24748307-20230614-01-bibr10] Chen , J. , & Wang , Y. ( 2021 ). Social media use for health purposes: Systematic review . *Journal of Medical Internet Research* , *23* ( 5 ), e17917 10.2196/17917 PMID: 33978589 PMC8156131

[x24748307-20230614-01-bibr11] Colineau , N. , & Paris , C. ( 2010 ). Talking about your health to strangers: understanding the use of online social networks by patients . *New Review of Hypermedia and Multimedia* , *16* ( 1–2 ), 141 – 160 10.1080/13614568.2010.496131

[x24748307-20230614-01-bibr12] Crozier , S. R. , Robinson , S. M. , Borland , S. E. , Godfrey , K. M. , Cooper , C. , Inskip , H. M. , & the SWS Study Group . ( 2009 ). Do women change their health behaviours in pregnancy? Findings from the Southampton Women's Survey . *Paediatric and Perinatal Epidemiology* , *23* ( 5 ), 446 – 453 10.1111/j.1365-3016.2009.01036.x PMID: 19689495 PMC3091015

[x24748307-20230614-01-bibr13] Dekker , R. L. , King , S. , & Lester , K. ( 2016 ). Social media and evidence-based maternity care: A cross-sectional survey study . *Journal of Perinatal Education* , *25* ( 2 ), 105 – 115 10.1891/1058-1243.25.2.105 PMID: 27445448 PMC4944453

[x24748307-20230614-01-bibr14] Fox , S. , & Duggan , M. ( 2013 ). Health online 2013 . *Health* , *2013* , 1 – 55 https://www.pewresearch.org/internet/2013/01/15/health-online-2013/

[x24748307-20230614-01-bibr15] Fryar , C. D. , Ostchega , Y. , Hales , C. M. , Zhang , G. , & Kruszon-Moran , D . ( 2017 ). *Hypertension prevalence and control among adults: United States, 2015–2016* . National Center for Health Statistics . https://www.cdc.gov/nchs/data/databriefs/db289.pdf 29155682

[x24748307-20230614-01-bibr16] Ghanbari , S. , Majlessi , F. , Ghaffari , M. , & Mahmoodi Majdabadi , M. ( 2012 ). Evaluation of health literacy of pregnant women in urban health centers of Shahid Beheshti Medical University. Daneshvar Medicine . *Basic and Clinical Research Journal* , *19* ( 6 ), 1 – 12 .

[x24748307-20230614-01-bibr17] Gilmour , J. A. ( 2007 ). Reducing disparities in the access and use of Internet health information. A discussion paper . *International Journal of Nursing Studies* , *44* ( 7 ), 1270 – 1278 10.1016/j.ijnurstu.2006.05.007 PMID: 16828775

[x24748307-20230614-01-bibr18] Hether , H. J. , Murphy , S. T. , & Valente , T. W. ( 2016 ). A social network analysis of supportive interactions on prenatal sites . *Digital Health* , *2* , 2055207616628700 10.1177/2055207616628700 PMID: 29942549 PMC6001212

[x24748307-20230614-01-bibr19] Howell , E. A. , Egorova , N. , Balbierz , A. , Zeitlin , J. , & Hebert , P. L. ( 2016 ). Black-white differences in severe maternal morbidity and site of care . *American Journal of Obstetrics and Gynecology* , *214* ( 1 ), 122.e1 – 122.e7 . 10.1016/j.ajog.2015.08.019 PMID: 26283457 PMC4698019

[x24748307-20230614-01-bibr20] Howell , E. A. ( 2018 ). Reducing disparities in severe maternal morbidity and mortality . *Clinical Obstetrics and Gynecology* , *61* ( 2 ), 387 – 399 10.1097/GRF.0000000000000349 PMID: 29346121 PMC5915910

[x24748307-20230614-01-bibr21] Huesch , M. D. , Galstyan , A. , Ong , M. K. , & Doctor , J. N. ( 2016 ). Using social media, online social networks, and internet search as platforms for public health interventions: A pilot study . *Health services research* , *51* ( Suppl. 2 ), 1273 – 1290 10.1111/1475-6773.12496 27161093 PMC4874940

[x24748307-20230614-01-bibr22] Kohan, S., Ghasemi, S., & Dodangeh, M. (2008). Associations between maternal health literacy and prenatal care and pregnancy outcome. *Iranian Journal of Nursing and Midwifery Research*, *12*(4).

[x24748307-20230614-01-bibr23] Kutner , M. , Greenburg , E. , Jin , Y. , & Paulsen , C . ( 2006 ). *The health literacy of America's adults: Results from the 2003 National Assessment of Adult Literacy* . National Center for Education Statistics . https://nces.ed.gov/pubs2006/2006483.pdf

[x24748307-20230614-01-bibr24] Manganello , J. A. , Falisi , A. L. , Roberts , K. J. , Smith , K. C. , & McKenzie , L. B. ( 2016 ). Pediatric injury information seeking for mothers with young children: The role of health literacy and ehealth literacy . *Journal of Communication in Healthcare* , *9* ( 3 ), 223 – 231 10.1080/17538068.2016.1192757 PMID: 29051785 PMC5645044

[x24748307-20230614-01-bibr25] Mellon , M. , Schiller , A. , Nelson , A. L. , & Stohl , H. E. ( 2020 ). Awareness of pregnancy-associated health risks among pregnant women and male partners surveyed in a prenatal clinic . *Journal of women's health (2002)* , *29* ( 3 ), 376 – 382 10.1089/jwh.2018.7585 31647358

[x24748307-20230614-01-bibr26] Moorhead , S. A. , Hazlett , D. E. , Harrison , L. , Carroll , J. K. , Irwin , A. , & Hoving , C. ( 2013 ). A new dimension of health care: Systematic review of the uses, benefits, and limitations of social media for health communication . *Journal of Medical Internet Research* , *15* ( 4 ), e85 10.2196/jmir.1933 PMID: 23615206 PMC3636326

[x24748307-20230614-01-bibr27] Morris , N. S. , MacLean , C. D. , Chew , L. D. , & Littenberg , B. ( 2006 ). The Single Item Literacy Screener: Evaluation of a brief instrument to identify limited reading ability . *BMC Family Practice* , *7* ( 1 ), 21 10.1186/1471-2296-7-21 PMID: 16563164 PMC1435902

[x24748307-20230614-01-bibr28] Nawabi , F. , Krebs , F. , Vennedey , V. , Shukri , A. , Lorenz , L. , & Stock , S. ( 2021 ). Health literacy in pregnant women: A systematic review . *International Journal of Environmental Research and Public Health* , *18* ( 7 ), 3847 10.3390/ijerph18073847 PMID: 33917631 PMC8038834

[x24748307-20230614-01-bibr29] Neighmond , P . ( 2019 ). *Why racial gaps in maternal mortality persist* . https://www.npr.org/sections/health-shots/2019/05/10/722143121/why-racial-gaps-in-maternal-mortality-persist

[x24748307-20230614-01-bibr30] Nielsen Company . ( 2017 ). *Reaching Black women across media platforms* . https://www.nielsen.com/us/en/insights/article/2017/reaching-black-women-across-media-platforms/

[x24748307-20230614-01-bibr31] Norman , C. D. , & Skinner , H. A. ( 2006 ). eHealth Literacy: Essential Skills for Consumer Health in a Networked World . *Journal of Medical Internet Research* , *8* ( 2 ), e9 – e9 10.2196/jmir.8.2.e9 PMID: 16867972 PMC1550701

[x24748307-20230614-01-bibr32] Ohnishi , M. , Nakamura , K. , & Takano , T. ( 2005 ). Improvement in maternal health literacy among pregnant women who did not complete compulsory education: Policy implications for community care services . *Health Policy (Amsterdam)* , *72* , 157 – 164 10.1016/j.healthpol.2004.11.007 PMID: 15802151

[x24748307-20230614-01-bibr33] Olander , E. K. , Smith , D. M. , & Darwin , Z. ( 2018 ). Health behaviour and pregnancy: A time for change . *Journal of Reproductive and Infant Psychology* , *36* ( 1 ), 1 – 3 10.1080/02646838.2018.1408965 PMID: 29517295

[x24748307-20230614-01-bibr34] Paige , S. R. , Krieger , J. L. , & Stellefson , M. L. ( 2017 ). The influence of ehealth literacy on perceived trust in online health communication channels and sources . *Journal of Health Communication* , *22* ( 1 ), 53 – 65 10.1080/10810730.2016.1250846 PMID: 28001489 PMC5551054

[x24748307-20230614-01-bibr35] Pew Research Center. (2021). *Social media fact sheet*. https://www.pewre-search.org/internet/fact-sheet/social-media/?menuItem=2fc5fff9-9899-4317-b786-9e0b60934bcf

[x24748307-20230614-01-bibr36] Renkert , S. , & Nutbeam , D. ( 2001 ). Opportunities to improve maternal health literacy through antenatal education: An exploratory study . *Health Promotion International* , *16* ( 4 ), 381 – 388 10.1093/heapro/16.4.381 11733456

[x24748307-20230614-01-bibr37] Sheinis , M. , Carpe , N. , Gold , S. , & Selk , A. ( 2018 ). Ignorance is bliss: women's knowledge regarding age-related pregnancy risks . *Journal of Obstetrics and Gynaecology: The Journal of the Institute of Obstetrics and Gynaecology* , *38* ( 3 ), 344 – 351 10.1080/01443615.2017.1357685 29022426

[x24748307-20230614-01-bibr38] Shieh , C. , Mays , R. , McDaniel , A. , & Yu , J. ( 2009 ). Health literacy and its association with the use of information sources and with barriers to information seeking in clinic-based pregnant women . *Health Care for Women International* , *30* ( 11 ), 971 – 988 10.1080/07399330903052152 PMID: 19809901

[x24748307-20230614-01-bibr39] Suarez-Lledo , V. , & Alvarez-Galvez , J. ( 2021 ). Prevalence of health misinformation on social media: Systematic review . *Journal of Medical Internet Research* , *23* ( 1 ), e17187 10.2196/17187 PMID: 33470931 PMC7857950

[x24748307-20230614-01-bibr40] Taheri , S. , Tavousi , M. , Momenimovahed , Z. , Direkvand-Moghadam , A. , Rezaei , N. , Sharifi , N. , & Taghizadeh , Z. ( 2021 ). Explaining the concept of maternal health information verification and assessment during pregnancy: A qualitative study . *BMC Pregnancy and Childbirth* , *21* ( 1 ), 252 10.1186/s12884-021-03715-7 PMID: 33771111 PMC7995715

[x24748307-20230614-01-bibr41] Yee , L. M. , Silver , R. , Haas , D. M. , Parry , S. , Mercer , B. M. , Wing , D. A. , Reddy , U. , Saade , G. R. , Simhan , H. , & Grobman , W. A. ( 2021 ). Association of health literacy among nulliparous individuals and maternal and neonatal outcomes . *JAMA Network Open* , *4* ( 9 ), e2122576 10.1001/jamanetworkopen.2021.22576 PMID: 34468757 PMC8411280

